# Meta-eQTL: a tool set for flexible eQTL meta-analysis

**DOI:** 10.1186/s12859-014-0392-0

**Published:** 2014-11-28

**Authors:** Antonio Fabio Di Narzo, Haoxiang Cheng, Jianwei Lu, Ke Hao

**Affiliations:** Department of Genetics and Genomic Sciences, Icahn School of Medicine at Mount Sinai, New York, NY USA; Icahn Institute of Genomics and Multiscale Biology Icahn School of Medicine at Mount Sinai, New York, NY USA; College of Electronics and Information Engineering, CIMS Research Center, Tongji University, Shanghai, China; School of Software Engineering and The Advanced Institute of Translational Medicine, Tongji University, Shanghai, China; Department of Respiratory Medicine, Shanghai Tenth People’s Hospital, Tongji University, Shanghai, China

## Abstract

**Background:**

Increasing number of eQTL (Expression Quantitative Trait Loci) datasets facilitate genetics and systems biology research. Meta-analysis tools are in need to jointly analyze datasets of same or similar issue types to improve statistical power especially in trans-eQTL mapping. Meta-analysis framework is also necessary for ChrX eQTL discovery.

**Results:**

We developed a novel tool, ***meta-eqtl***, for fast eQTL meta-analysis of arbitrary sample size and arbitrary number of datasets. Further, this tool accommodates versatile modeling, eg. non-parametric model and mixed effect models. In addition, ***meta-eqtl*** readily handles calculation of chrX eQTLs.

**Conclusions:**

We demonstrated and validated ***meta-eqtl*** as fast and comprehensive tool to meta-analyze multiple datasets and ChrX eQTL discovery. ***Meta-eqtl*** is a set of command line utilities written in R, with some computationally intensive parts written in C. The software runs on Linux platforms and is designed to intelligently adapt to high performance computing (HPC) cluster. We applied the novel tool to liver and adipose tissue data, and revealed eSNPs underlying diabetes GWAS loci.

## Background

Expression quantitative trait loci (eQTLs) are genomic loci that regulate expression levels of mRNAs, and eQTLs play important roles in genetics and systems biology studies. To date, multiple eQTL datasets (where both transcriptome and DNA genotype are profiled on the same individuals) exist for a given tissue type, e.g. liver and lung [1,2]. It is necessary to jointly analyze these sets to further improve statistical power especially for trans-eQTL discovery. Even for the same tissue type, the eQTL datasets (transcriptome and genotype data) could be heterogeneous due to platform and lab differences, and meta-analysis (but not pooled analysis) would be the method of choice. Meta-analysis is also desirable the analysis of chromosome X eQTLs in dataset consisting of both males and females. The interpretation of genotype effects on gene expression varies between genders. For example, an allele count of 1 in a female indicates a heterozygote genotype (one reference and one alternative allele), while a count of 1 in a male means only alternative allele exists and may cause more profound effects. The variance of the genetic effect may also differ between genders. In such scenario, directly pooling males and females in chromosome X eQTL discovery is invalid, while meta-eQTL tackles this issue elegantly by deriving eQTLs per gender and then combining the test statistics.

The typical strategy of meta-analysis has two steps: (1) calculate and record raw test statistics (e.g. *β* and pvalue) of every transcript-SNP pair per individual dataset, and (2) combine the statistics using meta-analysis approach. However, this strategy is not practical in eQTL setting, where each dataset requires evaluation of >10^11^ tests. Storing the raw statistics of every test is prohibitive due to massive disk and I/O demand. The common practice is only recording the top hits (e.g. pvalue < 1e-4) per dataset and meta-analysis. This strategy will miss the eQTLs that have consistent small-to-moderate effect in multiple datasets [3]. Herein, we propose the solution of parallel and synchronized eQTL computation of multiple datasets, and conducting meta-analysis on the fly. By these means, the above steps (1) and (2) are performed in memory, and only the meta-analysis results which pass a user-defined significance level are outputted to disk. Moreover, ***meta-eqtl*** offers versatile features: implementation of peak finding algorithm, various statistical models (eg. non-parametric and mixed effect model), consistent handling of missing data, easy deployment on high performance computing (HPC) clusters, *etc*.

## Implementation

***Meta-eqtl*** is a set of command line utilities written in R, with some computationally intensive parts written in C. Optimized linear algebra code (which is included in the R package) is used to fit linear models in absence of missing values. When missing values are present, in either the gene expression or SNP data, C code is called to compute the pairwise minimal sufficient statistics. The data format is based on plain text, tab-delimited files, which make the data easy to inspect and manipulate with standard UNIX utilities. Within ***meta-eqtl***, several modules are dedicated to specific functionality, and can be called individually by user.

Linear regressions meta-analysis is implemented in the R script ***lm-meta***. The computation occurs in multiple threads, where the number of threads corresponds to the number of datasets in the meta-analysis. The multiple threads proceed concordantly, therefore, the same set of gene expression-SNP tests are evaluated in each individual dataset at the same moment. When statistics are obtained from multiple threads, the meta-analytical test statistic is computed as:

$$ {\mathrm{Z}}_{\mathrm{meta}}=\left(\Sigma {\mathrm{w}}_{\mathrm{k}}{\mathrm{Z}}_{\mathrm{k}}\right)/{\left(\Sigma {{\mathrm{w}}_{\mathrm{k}}}^2\right)}^{1/2} $$

where the weights are assigned either based on sample size or the standard error of β in each dataset. The software output comprehensive statistics of the fitted model, including effective sample size, regression coefficients, standard error of regression coefficients, transcriptome variance explained (ie, r^2^), T statistics (***T***) and pvalues (***p***). The ***T*** and ***p*** were presented for both meta-analysis and each individual cohort. A separate utility, ***lm-fdr***, compares the output from observed and permuted data and quantify FDR. In brief, the meta-analysis results enter the downstream peak finding and empirical FDR calibration by permuting the sample IDs in the gene expression files. To our experience, this empirically estimated FDR is more robust than Benjamini-Hochberg procedure, such as used in MatrixEQTL [4], which is heavily biased when gene expression follows a non-Gaussian distribution. The tool set also contains the ***eqtl-sex-peaks*** utility, specially designed for meta-analysis of regression results by gender. ***kruskal*** is provided as a non-parametric Kruskal-Wallis test for eQTL detection. Since eQTL computation involves big data sets, gene expression and SNP data are accessed sequentially and concordantly by each thread, and results are reported on the fly, as they are computed. This allows for the analysis of files of arbitrary sample size and arbitrary number of datasets with constant memory usage. Also, this framework enables a natural deployment on HPC and Hadoop clusters as it can trivially distribute the analysis into multiple computing nodes.

## Results and discussions

To our knowledge, ***meta-eqtl*** is the first software to perform meta-analysis on arbitrary number of eQTL datasets. We thus compared our results with those obtained with METAL [5,6], a tool which performs meta-analysis on pre-stored test statistics. On a data of four individual sets (sample size of 1000, 1000, 500 and 500, respectively), we tested 10,000 SNPs, and the two software gave the identical results to the available numerical precision. We also benchmarked the performance on a large data of three cohorts (N = 450, 400 and 350) with 44,000 transcripts profiled and 1000 genome imputed genotype (~8 million SNPs). ***Meta-eqtl*** distributed the computation on a cluster of 800 computing cores (each core allocated 824 Mb to 1013 Mb of memory), and was able to complete within three days. The top eQTLs (10% FDR) statistics were identical to those computed by the R package “meta”. On a single cohort, we further conducted head-to-head comparison to the MatrixEQTL software [4], which to our knowledge is the fastest software available to date for large scale eQTL analysis, and found that ***meta-eqtl*** was about 2–3 times slower, reflecting the expense of missing data handling and the more flexible pipeline. Nevertheless, ***meta-eqtl*** is still one of the fastest eQTL tools available.

**Figure 1:**
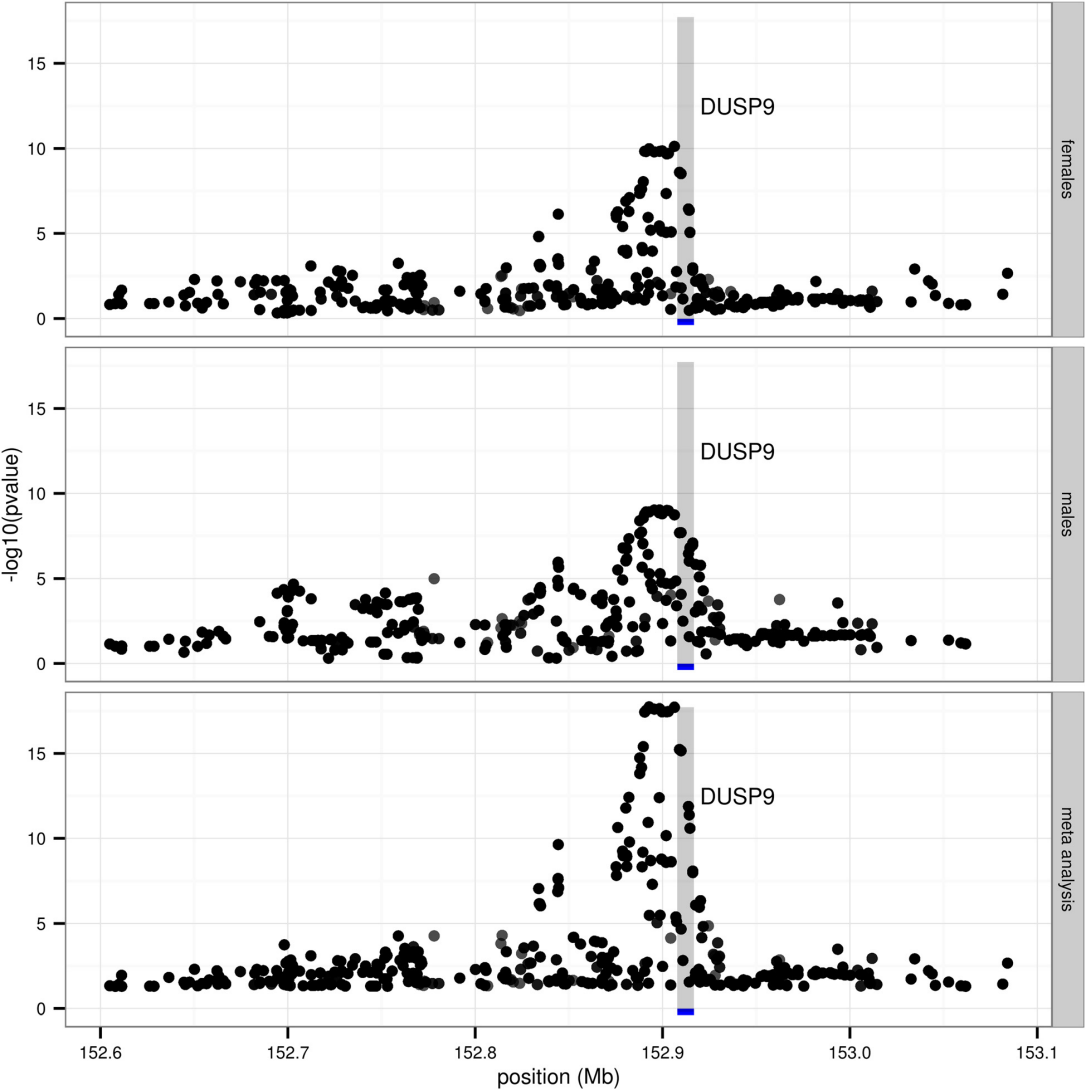
**Results of ChrX eQTL meta-analysis in Liver: cis-regulation of DUSP9.** Significance of association between genotype variants and DUSP9 expression. Position (in Mb) on the horizontal axis; −log10(pvalue) on the vertical axis. Top panel: associations in females; middle panel: associations in males; bottom panel: meta-analysis results. Highlighted in gray the position of DUSP9 on the genome. Although some evidence of cis-regulation of DUSP9 is already observed in males and females separately, the meta-analysis of the two datasets provides a sharper and more conclusive signal.

We also leverage another large-scale published eQTL study data [7], where custom 44 K RNA microarray were run on 651 liver, 848 adipose fat and 701 subcutaneous fat samples of 1,008 patients. 950 samples from the same patients were successfully genotyped on the Illumina 650Y BeadChip array, and further imputed on the 1000 Genome reference for 14 million SNPs using the MACH [8] pipeline. Applying meta-eQTL, we derived ChrX eQTL for each tissue (Table 1). The meta-analysis of males and females provides increased power in detecting genetic regulation of gene expression, while still correctly keeping separate the analysis of the two sets. In Figure 1, we illustrate e.g. how the X chromosome gene DUSP9 shows some evidence of cis-regulation in the liver of both females (top panel) and males (middle panel), with the meta-analytical results pointing to a sharper and more conclusive signal (bottom panel). Further, we employed the ChrX eQTLs to inform type 1 and type 2 diabetes (T1D and T2D) GWAS SNPs (where liver and adipose are disease relevant issues) documented in the NHGRI catalog [9]. Three chrX SNPs associated with T1D or T2D were also eQTLs in at least one of these three tissues (Table 2). Genes close to these SNPs were proposed as underlying the disease etiology in the original GWAS reports, herein, we identify additional plausible candidates (Table 3). For examples, rs2664170 is associated with T1D and has profound influence on gene expression levels of IKBKG in all tissues. IKBKG (inhibitor of nuclear factor kappa-B kinase subunit gamma) is the regulatory subunit of the inhibitor of IκB kinase (IKK) complex, which activates NF-κB resulting in activation of genes involved in inflammation, immunity, cell survival, and other pathways. Given the inflammatory basis of T1D, IKBKG is a highly relevant genetic risk factor. The direction of eQTL is consistent among the three tissues; that is the disease risk allele (rs2664170-G) is associated with lower level of IKBKG, leading to higher IκB kinase activity and elevated inflammation and in turn increase T1D risks.

**Table 1:** **Application of** ***meta-eqtl*** in meta-analysis of chrX eQTLs by gender*

**Tissue**	**#eQTL**	**# peak eSNP in RegDB**	**RegDB enrichment** ^**†**^	**# GWAS loci** ^**‡**^
**Liver**				
cis	131	80	2.36 (2.01 - 2.75)	34
trans	52	9	1.98 (1.17 - 3.16)	2
**Omental**				
cis	207	115	2.92 (2.56 -3.32)	54
trans	28	10	1.04 (0.64 -1.60)	0
**SubQ**				
cis	163	83	3.23 (2.81 - 3.72)	30
trans	18	8	1.39 (0.80 - 2.10)	1

*10% FDR eQTLs on chromosome X. Omental: omental fat tissue, *SubQ*: Subcutaneous fat tissue; ^†^Enrichment of ChrX eSNP in RegulomeDB database [10], odds ratio (95% confidence interval) are presented; ^‡^Number of ChrX eQTLs underlying GWAS loci.

**Table 2:** **ChrX diabetes GWAS hits with significant eQTLs in liver and adipose tissues**

**rsID**	**Position**	**Reported gene**	**Mapped gene**	**Disease**	**Risk Allele**	**GWAS pvalue**
**rs2664170**	153945602	Intergenic	GAB3	Type 1 diabetes	G	8e-9
**rs5945326**	152899922	DUSP9	KRT18P48 - DUSP9	Type 2 diabetes	A	7e-16
**rs12010175**	152862638	FAM58A	FAM58A	Type 2 diabetes	G	2e-9

**Table 3:** **eQTL statistics on ChrX diabetes GWAS hits**

**eQTL Gene**	eSNP rsID*	**eQTL Eff Allele**	**eQTL pvalue**	**β**	**T meta**	**T male**	**T female**	**eQTL Type**	**Tissue**
F8	rs2664170	A	3.8E-07	−3.60	−5.08	−2.93	−4.26	cis	Liver
AK095886	rs2664170	A	9.6E-04	−2.36	−3.30	−0.93	−3.43	cis	Liver
CTAG2	rs2664170	A	1.2E-03	2.35	3.25	0.69	3.46	cis	Liver
IKBKG	rs2664170	A	7.4E-03	1.92	2.68	1.07	2.53	cis	Liver
CTAG1B	rs2664170	A	8.7E-03	1.88	2.62	1.35	2.27	cis	Liver
AK095886	rs2664170	A	7.0E-07	−3.56	−4.96	−2.56	−4.34	cis	Omental
IKBKG	rs2664170	A	5.1E-05	2.90	4.05	2.48	3.26	cis	Omental
F8A1	rs2664170	A	2.4E-04	−2.61	−3.67	−1.53	−3.52	cis	Omental
SLC10A3	rs2664170	A	2.7E-04	2.59	3.64	2.99	2.30	cis	Omental
AK095886	rs2664170	A	8.1E-07	−3.52	−4.93	−2.88	−4.10	cis	SubQ
IKBKG	rs2664170	A	5.5E-04	2.45	3.46	3.35	1.77	cis	SubQ
Contig21200_RC	rs2664170	A	4.3E-08	−3.91	−5.48	−2.69	−4.96	trans	SubQ
XM_210086	rs5945326	A	7.0E-03	1.94	2.70	0.61	2.86	cis	Liver
SLC6A8	rs12010175	G	2.8E-04	2.62	3.63	2.99	2.41	cis	Liver
ARHGAP4	rs12010175	G	8.2E-03	1.91	2.64	0.13	3.08	cis	Liver
BC030106	rs12010175	G	1.8E-03	2.28	3.12	2.81	2.00	cis	Omental
HSS00085101	rs12010175	G	3.2E-03	−2.11	−2.94	−1.15	−2.82	cis	Omental
PLXNB3	rs12010175	G	3.0E-04	2.62	3.61	1.42	3.41	cis	SubQ
BC030106	rs12010175	G	7.6E-04	2.38	3.37	2.07	2.71	cis	SubQ

*SNPs rs2664170, rs5945326 and rs12010175 were identified as eQTL, and these SNPs are also reported in association with diabetes by large GWA studies (summarized in Table 2).

## Conclusions

In summary, we describe a novel package, ***meta-eqtl***. To our knowledge, it is the only tool to allow fast meta-analysis of eQTLs for today's large genotype and gene expression data with reasonable memory requirement and fast speed. It can also be used as a flexible and fast tool for eQTL discover on a single dataset, where it features flexible model specification (e.g. non-parametric and mixed effect models), missing data handling and implements significance peaks extraction. ***Meta-eqtl*** features computation speed comparable to the fastest alternative available to date, and is further well suited to distribute parallel jobs onto a HPC system. Another major advantage is the ability to handle chrX eQTLs. In recent year, increasing number of eQTL studies and dataset become available [3,7,11], joint analyses of same/similar tissue sets are of great interest. ***Meta-eqtl*** enables meta-analysis of arbitrary number of eQTL dataset and will greatly facilitate this research field.

## Availability and requirements

The ***meta-eQTL*** software package is freely available to all readers under https://haok01.u.hpc.mssm.edu/meta_eQTL/

**Project name:***meta-eQTL*

**Project home page:**https://haok01.u.hpc.mssm.edu/meta_eQTL/

**Operating system(s):** Linux

**Programming language:** R (version 3.0.2) and C

**License:** none

**Any restrictions to use by non-academics:** none
